# The Promises and Prospects of Long-acting Therapeutics for Treatment and Prevention of Infectious Diseases

**DOI:** 10.1146/annurev-pharmtox-071724-100739

**Published:** 2025-09-16

**Authors:** Adeniyi Olagunju, Simone Perazzolo, Zachary R. Stephen, Mark Ryan, Xiaolin Xu, Prajith Venkatasubramanian, Shakir Atoyebi, Rachele Delle Fratte, Andrew Owen, Charles Flexner, Rodney J.Y. Ho

**Affiliations:** 1 Centre of Excellence for Long-acting Therapeutics, University of Liverpool, United Kingdom; 2 Department of Biochemistry, Cell, and Systems Biology, University of Liverpool, United Kingdom; 3 Department of Pharmaceutics, University of Washington, Seattle, WA, USA; 4 Centre of Excellence for Long-acting Therapeutics (CELT), Department of Pharmacology and Therapeutics, University of Liverpool, United Kingdom; 5 Departments of Medicine and Pharmacology and Molecular Sciences, Johns Hopkins School of Medicine, Baltimore, MD, USA; 6 Department of Bioengineering, University of Washington, Seattle, WA, USA

**Keywords:** Long-acting medicines, Infectious diseases, Human immunodeficiency virus, Hepatitis, Tuberculosis, Malaria, Covid-19

## Abstract

Long-acting (LA) therapeutics have emerged as a key component of infectious disease treatment and prevention strategies, their uptake fueled by the need to bridge certain gaps notable with short-acting drug formulations. In this review, we present the key drivers and summarize the enabling technologies. Focusing on infections with significant global disease burden (HIV, hepatitis B and C, tuberculosis, malaria and COVID-19), the current state of knowledge on approved LA therapeutics and promising innovations currently in development are summarised. The potential role of LA therapeutics as countermeasures for diseases of pandemic potential as well as new approaches using computational modeling to accelerate their development for pediatric and perinatal health are discussed. Due to complexities in manufacturing, and the diversity of patent-protected technologies, barriers exist for global access to LA products and in upscaling intricate LA formulations. A multi-pronged strategic framework, including acceleration of equitable access through generic product manufacture, is proposed to realize the full potential of LA therapeutics for global health.

## Introduction

A long-acting (LA) therapeutic is a type of medication formulated to maintain effective drug concentration in the body for an extended period, allowing for less frequent dosing. Most oral medications in pill form maintain effective concentration in the body for only a few hours, necessitating daily dosing. While extended-release formulations can prolong this duration, they still generally require daily intake, with some exceptions, such as baloxavir marboxil with half-life exceeding 79 hours, allowing for single-dose oral treatment (1). Based on patient preferences and current clinical paradigms, general timescales considered for LA range from ≥1 week for oral pills to ≥1 month for parenteral, and ≥6 months for implants (2), but require indication-specific considerations. In the context of infectious diseases, LA modalities are transforming both treatment and prevention. Advances in formulation strategies enable simplified regimens, facilitate access and improve disease prevention and control.

For example, the approval of zidovudine and lamivudine in 1997 marked the first fixed-dose combination antiretroviral for HIV treatment. Subsequently, twice daily 3-drug fixed-dose combination of abacavir, lamivudine and zidovudine was approved in 2000, followed by once daily 3-drug fixed-dose combination of emtricitabine, tenofovir disoproxil fumarate and efavirenz in 2006. These improvements transformed HIV treatment strategies. From a disease that once ravaged whole communities, HIV is now a manageable chronic disease and patients have a near normal life expectancy with sustained viral suppression (3). The approval in 2022 of LA cabotegravir (CAB-LA) and rilpivirine (RPV-LA) administered 1-2 monthly, 6-monthly injectable lenacapavir (LEN) in 2023, and clinical development of a LA 3-drug regimen in a single injection intended to provide synchronized intracellular delivery to HIV host cells (Clinicaltrials.gov ID: NCT05850728) are unequivocal indications of further upcoming major transformations.

LA therapeutics have impacted several therapeutic areas. For example, LA contraceptives such as Depo-Provera have been in use since the 1970s. The low dose (~µg/day) requirement for hormonal contraceptives creates a perfect scenario for suitability with LA technology. Several LA drugs for mental health illnesses have also been used since late 1950s when fluphenazine decanoate was approved, with the most recent being the 2021 approval of a 6-monthly formulation of paliperidone palmitate (1.1-1.5g), an injectable second-generation antipsychotic. This approach has been shown to be transformative, resulting in reduced incidence of relapse, healthcare resource utilization, and cost (4, 5). Once-weekly injectable insulin products are similarly set to transform type 2 diabetes management, with evidence of greater improvement in long-term glucose control and time within normal range across seven randomised clinical trials (n = 3286, 60% on once-weekly injectable insulin) (6).

**Figure 1:**
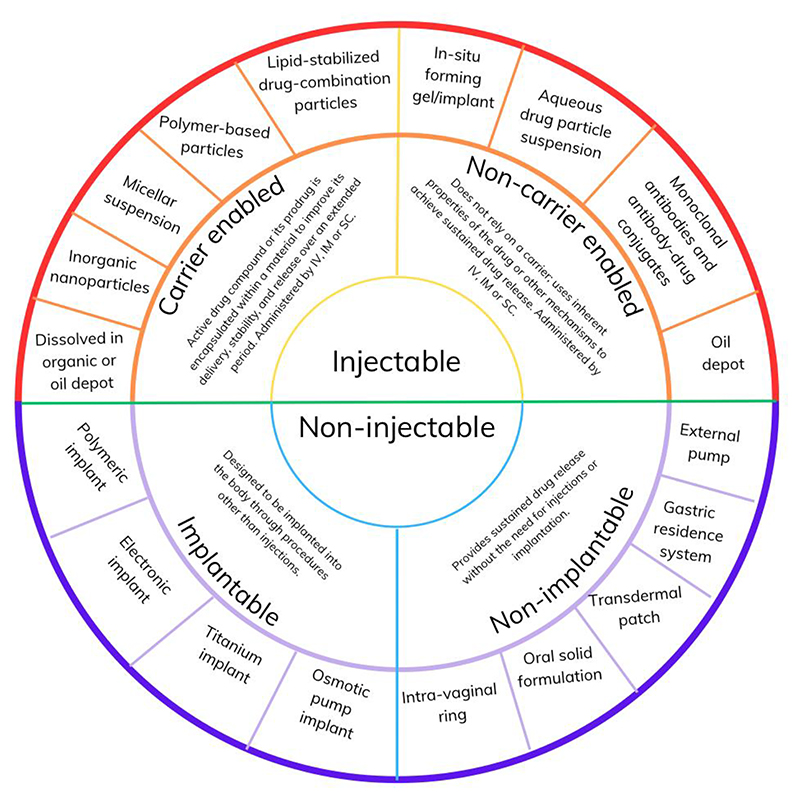
Enabling technologies for long-acting (LA) therapeutics. Most LA therapeutics currently approved or in development fall into one of two categories: injectable and non-injectable. In carrier enabled injectables, the active pharmaceutical ingredient is loaded within a drug delivery system that control release rate, compared with non-carrier enabled in which formulation characteristics of the free drug lead to LA drug delivery from the injection site. Non-injectable LA formulations are either implantable or non-implantable, the former being more invasive.

**Figure 2:**
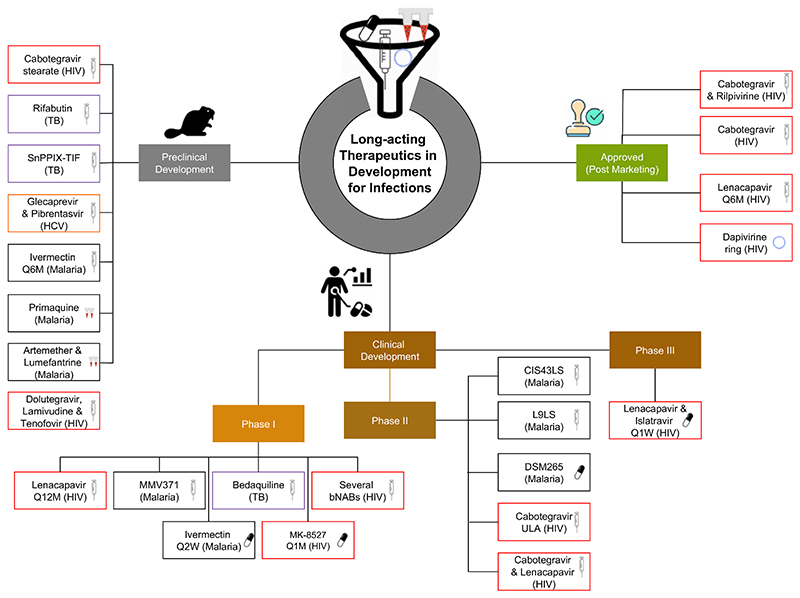
Examples of approved long-acting therapeutics and those currently in development (preclinical and clinical) for major infectious diseases. Indications are in brackets, each with different boundary color for distinction (red for HIV, human immunodeficiency virus; orange for HCV, hepatitis C; purple for TB, tuberculosis; black for malaria) and icons indicate the formulation type (e.g. needle and syringe for injectable, capsule for oral formulation). Wherever there are multiple development stages for a drug, the most advanced stage is indicated.

Are further transformations achievable in infectious diseases with the use of LA therapeutics? For instance, how close are we to having a one-shot LA therapy for: (1) hepatitis C cure administered same day as diagnosis; (2) all-season malaria prevention in endemic regions; (3) tuberculosis treatment or prevention in at risk individuals; or (4) or therapeutic for COVID-19 prevention to complement vaccination programs? In this review, we highlight key drivers and underlying technologies (Figure 1). The current state of knowledge on promising new approaches for treatment and prevention is summarised, focusing on infections with significant contribution to global disease burden (Table 1 and Figure 2), and promising applications of LA monoclonal antibodies (mAbs). Readers will also find coverage of additional topics helpful, including the role of *in silico* modeling in accelerating their development for pediatrics and perinatal health, potential utility of LA therapeutics in pandemic medical countermeasures, and the need for a framework to accelerate access through generic manufacture.

### Key Drivers for Long-acting Therapeutic Products

While one-pill-a-day oral medicines predominated new drug developments, the compelling rationale and market uptake of LA therapeutics is fueled by the need to bridge certain gaps notable with short-acting drug formulations. Some of these gaps include the following:

- **Adherence, convenience, and stigma:** Management of chronic illnesses is associated with significant daily (and multiple) pill burden that leads to suboptimal adherence (7), poor patient outcomes, and emergence of resistance in the case of infections like HIV and tuberculosis. Even the most potent and effective medications will fail if patients won’t take them due to inconvenience, misfit with patients’ often complex lifestyles (8), or stigma. In some cultures, there is significant social stigma associated with possession of prescription pills for some diseases.
- **Optimising treatment outcomes:** Maintenance of a specific drug concentration range throughout the dosing interval is critical for efficacy and safety. With traditional drug formulations, keeping trough (or minimum) drug concentration within this range often necessitates achieving relatively high peak concentrations (with higher doses) that exacerbate off-target effects. Wide daily peak-trough changes in concentration may be avoided with LA formulations that maintain the target therapeutic concentration, resulting in less sides effects and better symptom control.
- **Public health considerations:** Frequent clinic visits for daily oral medication prescription refills are associated with significant cost for patients and healthcare systems in settings with high disease burden and limited resources (9). The reduced dosing frequency of LA formulations can contribute to more effective healthcare delivery and better resource utilisation. For many LA formulations, lower doses are also required compared daily oral dosing when averaged across the dosing interval, potentially resulting in lower environmental exposure and lower bulk active pharmaceutical ingredient (API) costs.
- **Discovery of high potency APIs:** Increasing numbers of highly potent small molecules are becoming available, making drug delivery through LA formulation strategy possible with less frequent dosing.
- **Innovations in drug delivery technologies:** Recent advances in drug delivery technologies that support sustained and controlled release of drugs are enabling development of LA therapeutics.
- **Better option for prevention strategies:** The convenience, better adherence and avoidance of stigma associated with LA therapeutics make them ideal for prevention strategies in high-risk populations, providing a sustained level of protection (10).
- **Potential to align pediatric and adult formulations:** Suitability of certain LA formulation modalities across age groups could potentially remove the need to develop pediatric-specific formulations which often delay the introduction of new medicines in children. However, some technologies (e.g. microarray patches(11)) may be more suitable for LA drug delivery in pediatric populations rather than adults.

### Long-Acting Enabling Technologies

Small molecules (with molecular weight less than 1000 Dalton) constitute approximately 90% of the global pharmaceutical market and are leading in new drug approvals. They require daily dosing due to short biological half-lives (12). LA enabling technologies can extend half-lives of small molecule therapeutics with potential to bridge some of the gaps highlighted above, potentially improving adherence and health outcomes (13). In this section, we describe the technologies that enable LA drug delivery of small molecules.

Classified as injectable and non-injectable, general descriptions and examples are presented in Figure 1. Those with emerging or promising clinical applications for infections are subsequently described with relevant examples. Applications of LA technologies in peptide and protein-based therapies have been reviewed elsewhere (14).

1. **Oil depots:** These are concentrated formulation of drug administered by intramuscular injection to form a reservoir (or depot) at the injection site from which drug is gradually released into the bloodstream to provide sustained therapeutic effect. This first generation LA formulation strategy is simple to manufacture and administer. However, they are not compatible with many drugs, suffer from variable release kinetics, have dose limited injection volume, and cause injection site pain (2).
2. **Polymeric Microparticle Encapsulation:** Encapsulating polymers are biodegradable and provide greater control over drug release kinetics over several months. However, microparticle encapsulation technology is often limited by low drug encapsulation efficiency (15).
3. **Drug Particles in Aqueous Suspensions:** Over the last couple of decades, development of particle processing technologies (e.g. wet bead milling) that generate nanoscale drug particles in aqueous suspensions have enabled formulations with high drug loading (16). Drug nanoparticle formulations manufactured by wet bead milling tend to have high drug loads due to relatively low concentrations of surfactant needed to produce a stable product, and a high suspension strength avoiding loading based upon drug solubility. LA formulations of antiretrovirals CAB and RPV are manufactured this way (17), and other approaches to manufacturing of particle suspensions also exist (emulsion templated freeze/spray drying, nanoprecipitation, high pressure homogenization etc.). For example, the Unitaid-funded LONGEVITY project is utilising bottom-up solvent based approaches to formulate drug particle suspensions for HCV and latent tuberculosis infections.
4. ***In situ*-Forming Gels or Bioerodable Implants:** These are liquid polymeric formulations that solidify at body temperature (37°C) to form drug depots upon intramuscular or subcutaneous injection and provide sustained drug release (18). Various depot formation mechanisms have been used, including *in situ* precipitation, crosslinking, and solidification of thermo-reversable gels or assembly of liquid crystals (19). They are biodegradable, mitigating the need for implant removal.
5. **Subcutaneous Implants:** While inherently more invasive, they are highly promising for LA therapies and have already proven successful for contraception. Here the drug is imbedded in an inert polymer, or drug filled tubes designed for defined rate of release. They have the advantage of more consistent and predictable drug release when compared to intramuscular injections and provide the possibility of combining multiple drugs. The implant can be designed to be biodegradable or non-biodegradable, with the latter having the advantage of zero-order drug release kinetics leading to more consistent plasma levels (20). However, in addition to being more invasive, implants require insertion by someone else hence cannot be self-administered, non-biodegradable implants require surgical removal after drug depletion, and the overall drug dose possible is often lower.
6. **Microneedles assembled as microarray patches (MAPs):** These non-injectable, non-implantable drug delivery systems are minimally invasive. MAPs use drug loaded microneedles, each needle being up to 900 µm in length and each MAP carrying 25 needles/1.4x0.39 square centimeters (21). The drug loaded typically composed of inorganic, metallic, or dissolvable and degradable materials and are arranged onto a solid backed patch for drug delivery across the dermis of the skin. MAPs can be designed for the patch to be removeable after a short duration, the drug-loaded needles stay within the dermis to release drug over time (22). They have been tested to deliver drugs over several days and up to 2 months (23). While highly promising, it remains to be seen if MAPs will support adult dosing or multiple drug delivery due to their typically limited capacity (e.g. 2-3 mg per 16x16 or 19x19 MAP loaded with CAB and RPV). Conceptually, MAPs may be considered an alternative to needles and syringes, because LA formulation of the drug within the needle is often still required.
7. **Gastric resident systems (GRS):** GRS take advantage of the responsiveness of polymers to changes in microenvironment across the length of gastrointestinal system. The system resembles typical orally administered capsules. Once a GRS reaches the acidic environment in the stomach, extension of drug loaded polymeric arms prevents progression into the intestine, releasing its payload in the stomach through gradual erosion of the arms. The extended drug release provides plasma drug levels for about a week, overcoming daily dosing requirement due to GI transit time of 24 hr, after which the system disintegrates and is expelled through GI transit. The multiple drug-eluting arms may allow the system to support combination therapy (24).
8. **Drug Combination Nanoparticulate (DcNP) Systems:** DcNP take advantage of the ability of amphipathic properties of lipid excipients to assemble drugs with disparate water solubility profiles into particles that are stable in storage and in the body (25, 26). This technology supports coformulation of LA therapies in one injectable, with synchronized drug delivery in lymphocytes. Pioneered under the University of Washington NIH and Unitaid-funded Targeted, Long-acting and Combination Anti-Retroviral Therapy (TLC-ART) program, the DcNP technology has been applied to multiple antiretroviral drugs.

The Long-acting Therapeutics Patents and Licences Database (LAPaL, https://lapal.medicinespatentpool.org/) provides access to carefully curated information on available LA technologies and molecules across multiple therapeutic areas, including visualisation tools to explore their global clinical development landscape and regulatory approval status.

### Mechanisms of Long-acting Drug Delivery Systems

A detailed discussion on how the different technologies cited above extend drug exposure and duration of action is beyond the scope of this review. However, release-dependent half-life extension is a major mechanism in LA therapeutics currently in clinical use. Examples include: (1) drug particle suspensions where the slow rate of dissolution controls drug absorption; (2) prodrugging where an additional hydrolytic step is postulated to slow down absorption; and (3) encapsulation that may slow down absorption by restricting drug release from the carrier and/or force absorption through the lymphatic system which also slows down entry to the systemic circulation. Importantly, some technologies may be driven by multiple mechanisms.

### Long-Acting Therapeutics for Global Health Challenges

#### HIV treatment and prevention

Based on 2023 estimates, about 39.9 million people globally live with HIV, a virus that continues to pose a significant public health challenge despite four decades of research (27). One of the most significant advances in HIV treatment is the concept of Undetectable=Untransmittable (U=U), to emphasise that individuals with an undetectable viral load cannot sexually transmit the virus. However, traditional HIV treatment with antiretroviral therapy (ART) involves lifelong daily oral medication, often consisting of three drugs (28). This daily regimen can lead to pill fatigue, poor adherence, and the need for more complex regimens in case of viral rebound and drug resistance. Furthermore, the costs and logistics of delivering prescription refills for daily oral ART, especially in rural settings, impose additional burdens on healthcare systems and patients. LA ART with less frequent dosing, such as monthly or bi-monthly injections, could alleviate many of the challenges associated with daily oral ART. Surveys among people living with HIV show a preference for LA over daily oral treatments (29).

Since December 2021, a few LA products have been approved for HIV treatment and/or prevention (Table 1 and Figure 2). The dual injectable CAB/RPV-LA is a complete regimen approved as maintenance therapy in adults and adolescents who are stable on ART with an undetectable viral load. It includes injectable aqueous drug particle suspensions of CAB and RPV, given intramuscularly every 1 or 2 months. It was shown to be as effective as daily oral ART in maintaining viral suppression in multiple clinical trials, including LATTE (30), ATLAS (31), FLAIR (32), ATLAS-2M (33). CAB-LA is now also approved for HIV pre-exposure prophylaxis (PrEP) in high-risk populations after results from trials like HPTN 083 (34) and HPTN 084 (35) demonstrated that CAB-LA was superior to daily oral emtricitabine-tenofovir disoproxil fumarate in preventing HIV transmission.

LEN, a first-in-class capsid inhibitor, is approved for treating multi-drug-resistant HIV in combination with other antiretrovirals. LEN exhibits LA pharmacokinetics when its solution is administered subcutaneously every six months. The CAPELLA trial confirmed greater reduction from baseline in viral load (primary endpoint: at least 0.5 log_10_ copies per milliliter by day 15) was achieved in 88% LEN-treated patients compared with 17% of those who received placebo (36). In the CALIBRATE trial, high virological suppression rate was achieved by week 4 in treatment-naive individuals (81-87%) who received 6-monthly LEN in combination with other ART regimens, further increasing by week 26 (37). Two large PrEP studies compared 6-monthly LEN with daily oral emtricitabine-tenofovir disoproxil fumarate in reducing the risk of HIV transmission. There were 0/5338 infections in PURPOSE 1 trial with incidence rate ratio (IRR) of 0.00 (95% CI, 0.00 to 0.10, P<0.001); and 2/3265 infections in PURPOSE 2 trial (IRR = 0.11, 95% CI, 0.02 to 0.51, p = 0.002), which is unprecedented in the HIV PrEP field to date (38, 39). A dapivirine-loaded silicone matrix vaginal ring that releases the antiretroviral drug over 28 days was approved in multiple African countries after it was shown to reduce HIV incidence by 30% among women in PrEP clinical trials (40, 41).

Emerging innovations include DcNP system, developed under the University of Washington NIH and Unitaid-funded TLC-ART program. It exhibits LA pharmacokinetics in non-human primates (42) and has been used to co-deliver lipophilic and hydrophilic drugs e.g. tenofovir, lamivudine and dolutegravir (26); lopinavir, efavirenz, and tenofovir (43); atazanavir, ritonavir, and tenofovir (44). DcNP exhibits lozenge-shape nanostructure, and was shown to be readily and preferentially taken up into the lymph capillary, rather than blood, after subcutaneous dosing and widely distributed throughout the lymphatic system (45). Higher cellular drug levels are noted in the cells of the nodes compared to those in the blood, which is estimated to be higher than in plasma (25, 26, 46). One of the DcNP-enabled products, TLC-ART 101, containing water-insoluble lopinavir, ritonavir and water soluble tenofovir in a single subcutaneous injection has completed a proof-of-concept phase 1 safety and pharmacokinetic study (47). The DcNP-based platform was recently leveraged to extend the exposure of all three components of a first-line antiretroviral regimen (dolutegravir, lamivudine and tenofovir) above predicted viral-effective concentrations for up to 4 weeks in non-human primates (26). Several LA broadly neutralizing antibodies (bNAbs) are currently in development for HIV treatment and/or prevention, paired with other bNAbs, ART, or latency-reversing agents. Comprehensive reviews of LA bNAbs were recently published by other authors focusing on HIV prevention (48), treatment trials (49) and pediatric trials (50).

Islatravir is a first-in-class nucleoside reverse transcriptase translocation inhibitor (NRTTI) with multiple mechanisms of action and extended half-life amenable to LA therapy. Multiple NRTTIs are currently in clinical development, including islatravir (0.25 mg) in combination with LEN as a once weekly oral tablet in phase 3 and MK-8527 for monthly oral PrEP in phase 1 (Table 1, Figure 2). Clinical development of MK-8507 was suspended due to CD4+ T cells decline greatest in those who received it in combination with 0.75 mg islatravir once-weekly. Some promising LA modalities in development aim to achieve ultra-long acting delivery similar to LEN, including ULA CAB with potential for 4-monthly administration in phase 2 (Table 1).

Advancing LA technologies and therapeutics for HIV treatment and PrEP currently in preclinical stage to clinical development is not only expected to generate additional options in the coming years, but also fill important gaps. Examples include tunable biodegradable and removable implants capable of maintaining effective drug levels for up to a year in animal models (51), particle suspensions for novel prodrugs of water soluble nucleoside reverse transcriptase inhibitors (52, 53),and ULA dolutegravir prodrug homodimer shown to sustain delivery up to four months (54).

LA therapies represent a frontier in HIV treatment and prevention, offering hope for more convenient and effective care, with high patient acceptability and satisfaction. However, there are challenges to its widespread adoption, including injection site reactions, need for clinic visits due to lack of self-administered options, high costs, and ongoing limited access in low- and middle-income countries due to patent restrictions (55, 56). Requirement for in-clinic administration poses logistical challenges, particularly in regions with limited access to healthcare facilities, precipitating concerns regarding potential risk of drug resistance developing in patients who may fail to turn up on time for their repeat injections. Emerging evidence indicates low incidence of virological failure in patients receiving CAB/RPV-LA (1% in induction-maintenance and switch-suppressed studies, 5% in switch-viraemic studies).

However, integrase strand transfer inhibitor resistance emerged in 40-70% of virological failure cases in patients who received CAB/RPV-LA, much higher than observed with oral integrase strand transfer inhibitor based regimen (57).

Ongoing trials to evaluate LA ART in special populations, such as pregnant women (CAB/RPV-LA in CREATE study, Clinicaltrials.gov ID: NCT06336434), lactating women (MK-8527 clinical lactation study, NCT06580587), and children from 2 to < 12 years (CAB/RPV-LA in CRAYON study, NCT05660980), are expected to highlight any special considerations that may be warranted in these populations.

#### Long-acting therapeutics for hepatitis B & C

About 354 million people live with hepatitis B (HBV) and hepatitis C (HCV) virus infections globally, resulting in 940,000 annual deaths (58). This is despite an effective universal childhood immunization for hepatitis B prevention and control. Up to 25% of individuals with chronic HBV infection require lifetime treatment to avoid progression to cirrhosis or liver cancer. However, access to testing and treatment is limited in low- and middle-income countries (59). Approved treatment protocols for HBV consist of daily oral antivirals: entecavir, tenofovir dipovoxil fumarate, and tenofovir alafenamide. A weekly subcutaneous injection of Peg-interferon alpha is also available (60).

On the other hand, there is currently no approved vaccine against HCV. Direct-acting antiviral (DAAs) drugs are used for treatment and cure rates over 95% have been achieved. However, country-level data on disease burden and access to diagnosis and treatment results in a 55-85% progression to chronic disease stage (61, 62). HCV treatment regimens include daily oral dosing of a combination of at least two pan-genotypic DAAs with different mechanisms of action. Approved curative regimens include sofosbuvir plus velpatasvir, glecaprevir plus pibrentasvir, sofosbuvir and daclatasvir and sofosbuvir plus velpatasvir plus voxilaprevir. Most people achieve cure with 8-12 weeks of treatment, defined as plasma HCV RNA less than the lower limit of quantification 12 weeks post therapy without prior failure. There is emerging evidence of maintenance of high cure rates with 4-or 8-week shortened sofosbuvir and daclatasvir therapy instead of 12 weeks, with retreatment if needed (63).

The high pill burden and requirement for multiple clinic visits is incompatible with the often-complicated lifestyle of those affected, potentially leading to suboptimal adherence. As recently reviewed by Thomas *et al* (64), available NRTI for HBV and DAAs for HCV are promising candidates for LA strategy. Multiple LA formulations of tenofovir and its prodrug tenofovir alafenamide are currently in development, including subdermal implants. A bioinert titanium–silicon carbide nanofluidic implant provided sustained tenofovir alafenamide delivery (65). A phase 1/2 study (CAPRISA 018) designed to evaluate it in young women was suspended due to intolerable local reaction (66).

About 90% of new chronic HBV infections are acquired perinatally and 90% progress to chronic infection (67). Hence, The World Health Organization (WHO) now recommends that all HBV positive pregnant women receive tenofovir prophylaxis starting from the 28th week of pregnancy until at least birth to prevent mother-to-child transmission (68). A single dose LA formulation of an existing anti-HBV drug administered during the second or third trimester for prevention of mother-to-child transmission would positively impact these efforts. On the other hand, combining point-of-care HCV diagnosis with curative LA DAA could enable a one visit test-and-cure protocol that would transform HCV treatment landscape and open up a pathway towards its elimination. A retrievable gastric resident system for LA delivery of sofosbuvir for HCV treatment provided a month-long drug release in a swine model (69). While the nasogastric route of administration may not find widespread clinical application, the demonstrated cost effectiveness across various patient populations illustrates the potential public health impact of LA strategy for HCV therapeutics. Finally, a glecaprevir / pibrentasvir combination aqueous particle suspension LA injectable (LAI) is under development as part of the Unitaid-funded LONGEVITY project with extremely promising preclinical data, and is currently moving towards phase 1 evaluation (Table 1 and Figure 2).

#### Long-acting treatment and prevention options in development for tuberculosis

Global TB incidence rate was estimated to be 133 new cases per 100,000 population per year with 410,000 multidrug-resistant TB (MDR TB) cases in 2022 alone (70). Among low-income countries, the Philippines had the highest prevalence, exceeding 500 cases per 100,000 people and notably higher among vulnerable populations, such as indigenous communities and those exposed to air pollution (71). Pharmacotherapeutic management depends on whether the infection is drug susceptible, drug-resistant, or MDR TB, the standard duration being 4-6 months. This can extend to between 9 and 20 months with oral and injectable combination therapy depending on severity and resistance pattern (70, 72). As of August 2023, 28 novel drugs being tested for TB treatment were in various phases of clinical trials including compounds like BVL-GSK098 (Bioversys) and BTZ-043 (University of Munich). Three new drugs (bedaquiline, delamanid and pretomanid) and 7 repurposed drugs (including clofazimine and levofloxacin) were recently recommednded by WHO (70). In line with current guidelines, US Centers for Disease Control and Prevention (CDC) recommends the use of once-weekly isoniazid and rifapentine for 12 weeks to treat latent TB infection in children aged 2 to 17 years old. Similar treatment is recommended in patients co-infected with HIV (73).

A Phase 1 clinical trial is currently underway for intramuscular long-acting injectable (LAI) formulation of bedaquiline (a diarylquinoline) in healthy volunteers (74). Bedaquiline encapsulated in novel nanocarriers was reported to release the drug over 7 days, depending on the type of nanocarrier and the medium used (75). Promising results were reported for LAI rifabutin composed of poly(lactic-co-glycolic acid) and dimethyl sulfoxide in preclinical studies, supporting up to 16 weeks drug release in mice (76). Several inhibitors of membrane protein large 3 (MmpL3), crucial for cell wall synthesis in *Mtb* through its effects on trehalose monomycolates transport across the inner membrane (77), are in development (78). Niosomes loaded MmpL3 inhibitor BM859 was reported to exhibit potent antimycobacterial activity against *Mtb*, with significantly loading capacity and sustained drug release over 60 days in mice (79). Once-weekly injection of a poloxamer-poly(acrylic acid)-based thermoresponsive injectable formulation of tin protoporphyrin IX, a heme oxygenase-1 inhibitor with anti-*Mtb* activity and treatment shortening capabilities as a host-directed therapy in mice, demonstrated higher AUC_0-24_ and C_max_ compared with daily dose of the free drug in a proof-of-concept study (80). A rifapentine aqueous particle suspension LAI is under development at CELT as part of the Unitaid-funded LONGEVITY project with extremely promising preclinical data, and is currently moving towards phase 1 evaluation. The team at the Centre of Excellence in Long-acting Therapeutics (CELT) have also generated preclinical proof-of-concept for a particle suspension LAI containing TBAJ-876 (Table 1 and Figure 2).

A comprehensive review highlights promising drug delivery systems for TB management, including lipid nanoparticles, polymer nanoparticles, inorganic nanoparticles, emulsion-based systems, carbon nanotubes, graphene, and hydrogels (81); many of these enable LA strategy.

Long plasma half-life, poor water solubility, and high potency (to facilitate reasonable injection volume) of drug candidates in development for TB are important components of target product profile recommended for compatibility with LA strategy (82). Based on these attributes, achievable LA potential could be projected using physiologically-based pharmacokinetic (PBPK) modelling. For example, monthly intramuscular dose of 1500 mg delamanid combined with 250 mg rifapentine was predicted to provide therapeutic exposures for TB treatment using this approach (83).

#### Are LA antimalarials on the horizon?

Despite reduction in overall mortality from 29 per 100,000 at risk individuals in year 2000 to 14.3 per 100,000 in 2022, malaria remains an ongoing public health challenge in endemic countries. The WHO estimates that 249 million infections and 608,000 deaths were attributable to malaria in 2022, with approximately 76% of deaths occurring in children under five years of age (84).

Currently, effective malaria treatment and chemoprevention is achieved through routine oral-dosing regimens (85). Recommendations for include intermittent preventive treatment of malaria in pregnancy, perennial malaria chemoprevention (previously known as intermittent preventive treatment in infants), seasonal malaria chemoprevention, intermittent preventive treatment in school aged children, post-discharge malaria chemoprevention and mass drug administration for malaria burden and transmission reduction, and mass relapse prevention. However, daily oral dosing for chemoprevention and antimalarial treatment schedules are associated with non-adherence (86, 87).

LA formulations of effective antimalaria drugs are expected to make existing malaria management and elimination strategies more efficient, potentially making the WHO Assembly target of 90% reduction in malaria mortality and infections by 2030 more achievable (88, 89). Aspirational target product profile attributes for a LA antimalaria therapy include suitability for all age groups, 3-4 monthly (or monthly oral) dosing frequency with over 80% protection, activity against blood stage parasite, less than $5 and no requirement for cold storage (90). The first proof-of-concept for a LA injectable for malaria chemoprophylaxis was published in 2018 (91). LA formulations of several investigational or repurposed compounds are now in development for malaria treatment and prevention (90).

Ivermectin is a broad-spectrum anthelmintic that possesses significant potential as a first-in-class endectocide providing vector-based malaria control (92). An extended-release oral ivermectin formulation coded LYN-163 is currently in phase 1 clinical trials. This oral drug delivery system is based on a proprietary gastric resident system technology which supports once fortnightly dosing (93). Other LA ivermectin formulations are in pre-clinical development, including a polymer-based injectable drug delivery system (94). DSM265 is a once-weekly orally active compound that functions by selectively inhibiting the plasmodium dihydroorotate dehydrogenase enzyme essential for *P. falciparum* pyrimidine biosynthesis. While initial preclinical studies indicated promise, subsequent in-human clinical trials showed that DSM265 induces limited pre-erythrocytic protection, indicating that alternative dosing regimens or combination therapy approaches may be necessary (95).

CIS43LS and L9LS are mAbs in phase 2 clinical development that offer additional methods of passive malaria prophylaxis. These mAbs target highly conserved regions of the circumsporozoite protein essential for *P. falciparum* infection. L9LS selectively binds the minor NVDP repeats, a specific sequence of amino acids in the circumsporozoite protein, while CIS43LS possesses specificity for the junctional NPDP epitope, another important sequence that plays a role in the parasite’s ability to infect its host. Site-directed mutagenesis within the fragment crystallizable (Fc) region extends their longevity and half-life, with CIS43LS displaying high levels (88.2%) of malaria efficacy for 24 weeks following a single intravenous infusion. These LA protective titers highlight their potential as single-dose chemoprophylaxis for seasonal malaria in high-transmission endemic regions (96, 97).

Multiple investigational LA compounds are also in preclinical development for malaria treatment and seasonal chemoprophylaxis (Table 1 and Figure 2), including P218, an injectable formulation of *P. falciparum* dihydrofolate reductase inhibitor. P218 displays strong activity against pyrimethamine-resistant malaria, affecting both blood and liver-stage parasites (98). An intradermal hydrogel dissolving microarray patch (MAP) was evaluated for the delivery of chloroquine and primaquine in a preclinical study, with sustained plasma levels observed for both drugs over 7 days in rats. Efficacy studies in a murine malaria model showed up to 99.2% reduction in parasitaemia (99). The target product profile for the primaquine MAP includes a wear time and administration frequency for primaquine of a single MAP over 2 weeks (optimal), or replaced every 3 days (minimal) (100). This delivery system was similarly evaluated for artemether and lumefantrine, using freeze-dried nanosuspensions of both drugs (101). Others include LAI formulations of atovaquone with potential for 1 monthly intramuscular administration (91), as well as LAI formulations of proguanil, piperaquine and pyronaridine (88). An important consideration for these LA formulations to have a lasting impact on malaria treatment and prevention programmes will be the potential to be paired with another LA agent as a complete regimen.

#### Long-acting monoclonal antibodies

The high binding site specificity of mAbs presents advantages over small molecule therapeutics including fewer off-target effects and drug-drug interactions, and lower toxicity (102). Broadly neutralizing antibodies (bNAbs) provide additional advantages due to their ability to neutralize a wide range of viral variants and are promising as LA therapeutics. Most mAbs administered intravenously demonstrate a half-life that allows dosing every 4 weeks, with potential for 3-6 months through Fc domain modifications that enhance the binding of the neonatal Fc receptor (FcRn), an important factor in the serum half-life of immunoglobin G. FcRn binding can be enhanced through various modifications to the Fc domain, with YTE modification (M252Y, S254T, and T256E) and LS modification (M428L and N434S) most commonly utilized (103). mAbs are approved or currently in development for a range of infectious disease. Palivizumab and nirsevimab are approved for respiratory syncytial virus infection prevention in high-risk infants, and raxibacumab and obiltoxaximab are approved for prophylaxis and treatment of anthrax. The half-life of palivizumab is approximately 480 hours (104, 105), while the extended half-life of nirsevimab provides protection for at least 5 months through YTE modification (106). (107) Numerous mAb products are currently in development for HIV and COVID-19 (108). In the case for HIV, antibodies have been widely ineffective in controlling the viral load, largely due to the heterogeneity of HIV-1 variants. Therefore, several strategies utilizing bNAbs are being explored. Ibalizumab was able to achieve virologic suppression in patients with repeated episodes of virologic failure due to multidrug-resistant HIV or drug intolerance in phase 3 clinical trials (109). However, subsequent virological failure was observed in some patients due to diminished ibalizumab susceptibility. Combination therapy using two or more bNAbs may help address transient suppression of viraemia and emergence of resistant strains associated with monotherapy. For instance, combinations of two bNabs was shown to be effective in maintaining suppression for extended periods in individuals harboring HIV-1 strains sensitive to the antibodies (110, 111). Another promising strategy is the development of regimens that combine bNAbs with potent antiretrovirals to minimize the risk of resistant variants emerging from viral reservoirs (112). Yet successful application of multiple bNAb combination therapy or pairing of bNAbs with LA ART must overcome several challenges including alignment of dosing intervals, the need to administer bNAbs by infusion, and continued concerns over development of resistant strains (111–113).

The emergence of SARS-CoV-2 highlighted the challenges of developing mAb therapies against rapidly changing infectious diseases. Several mAbs were granted early emergency use authorization due to their effectiveness in reducing SARS-CoV-2 RNA, including a combination of bamblanivimab and etesevimab (114). However, the emergence of SARS-CoV-2 variants with reduced susceptibility upended their use in most healthcare systems. Similar challenges arose for the use of mAbs for prevention (115). Despite these challenges, there are reasons to remain positive. Two emerging extended half-life (LS modification) mAbs, AER001 and AER002, have shown to be well tolerated and demonstrated high serum neutralization against D614G and Omicron BA.1 in clinical trials (116). However, long-term effectiveness with newly emerging variants remains a concern.

#### Future Outlook

Currently, over 90% of approved drugs have inadequate safety recommendations to guide use during human pregnancy or lactation (117). This results from the long-standing practice of excluding these populations from clinical trials (118) due to real (and sometimes imagined) safety, ethical and legal concerns (117, 118). Off-label prescription of many drugs for pregnant and breastfeeding individuals are based on approved dose in non-pregnant adults, while weight adjusted dosing is used for children (118, 119). This practice comes with risk of toxicity or suboptimal exposure due to well established physiological changes during the perinatal period, and rapid developmental changes in metabolic function in children (119, 120).

PBPK modeling is a highly adaptable, mechanistic pharmacometrics approach used for whole-body prediction of drug disposition over time. Generally, these models employ mathematical equations to describe the time-course of body-drug interaction and can be adapted to include changes in physiology seen during gestation, child development, and LA formulation characteristics. Hence, they are capable of bridging the existing gaps in our understanding of LA therapeutics disposition in women during the perinatal period, and in children (121). This includes ahead of the availability of clinical pharmacokinetic data and to guide decisions about the LA potential of available dose selection (122, 123). At the time of writing this review, 22 such models have been published for LA therapeutics, covering scenarios ranging from investigation of LA potential of small molecule drugs, characterisation of variables that affect LA drug disposition, dose selection for humans, drug-drug interaction liability and dose adjustment amongst others (122–143). Only three of these published PBPK models involved pregnant women (123) or children (122, 133) in the studied populations. CELT developed a free-to-access online PBPK tool named Teoreler to broaden availability of this approach to global investigators (https://www.liverpool.ac.uk/centre-of-excellence-for-long-acting-therapeutics/teoreler/). There is currently no published lactation PBPK model for a LA therapeutic.

In one published PBPK study, monthly and two-monthly dosing regimens of injectable CAB-RPV LA initiated during pregnancy was investigated (123). Despite adequate CAB exposure throughout pregnancy, RPV plasma concentration was predicted to be suboptimal during pregnancy when administered monthly or two-monthly. These predictions were confirmed by another group in a single pregnant patient receiving the bimonthly dosing regimen of CAB-RPV LA during third trimester of pregnancy (123, 144). A Framework for Dose Selection in Pregnancy to support clinical implementation of model-informed doses during pregnancy was recently developed (145), trialled with antenatal dosing strategy for sertraline in anxiety disorders and depression. PBPK modeling was recently used to support drug label information for oral moxidectin during breastfeeding (146). Adoption of similar approaches for LA therapeutics will be an excellent way to promote evidence-based dosage recommendations for their use in perinatal and pediatric health.

Regulatory support and guidance for the use of adequately qualified PBPK models has facilitated increase in the inclusion of data generated by this approach in successful drug approval filings (147–149). The 2023 ICH E21 concept paper on the inclusion of pregnant and breast-feeding individuals in clinical trials highlights potential use of PBPK models to inform participation of pregnant and breastfeeding individuals in clinical trials (150). Ultimately, using this approach for LA therapeutics is likely to increase efficiency and accelerate their introduction in women during the perinatal period and in children.

The change in COVID-19 pandemic trajectory due to the availability of effective mRNA vaccines against SAR-CoV-2 highlights the importance of vaccination programs. However, certain persistent issues emphasize the need for drug therapy options as part of medical countermeasures in pandemic preparedness: (1) emergence of variants (e.g. due to changes in SAR-CoV-2 spike protein); (2) contraindications (e.g. in persons with anaphylactic reaction, or poor vaccine response); and (3) vaccine hesitancy across different populations. Three approved small molecule antiviral drugs that target sites other than the spike protein, molnupiravir, nirmatrelvir and ritonavir, and remdesivir, have been shown to be effective across different SARS-CoV-2 variants (151). LA formulations of broad-spectrum antiviral agents would be invaluable medical countermeasures against infections with pandemic potential like COVID-19. This could enable one-shot prevention or treatment for an extended period before a suitable vaccine candidate becomes available, and when vaccines cannot be used. In this regard, the anti-SARS-CoV-2 activity of repurposed niclosamide has been demonstrated in multiple studies (152–154). Its ambient temperature-stable particle formulation stored as solid for reconstitution in aqueous vehicle when needed, provided extended drug exposure for 1 month in preclinical animal model (155). The clinical efficacy of niclosamide for SARS-CoV-2 is highly uncertain, but this work highlights how LAI formulation strategies could be applied to broad spectrum antiviral drugs in preparation for future outbreaks.

## Conclusions

To realise the full potential of LA therapeutics for infections of global health importance, a strategic framework to guarantee equity in access, especially in low- and middle-income countries (LMICs) where the products are most needed due to high disease burden, will positively impact global health. This may include:

1. Enabling early entry of generic manufacturers to facilitate global access. This requires facilitating licence agreement between innovator companies and generic manufacturers. The Medicines Patent Pool has led the charge to develop voluntary licensing model which has facilitated the delivery of over 40 billion doses of medicine in LMIC, saving US$ 2 billion in global health funding. Three sublicences for CAB-LA for HIV PrEP were signed in 2022 with three generic manufacturers to enable access in 90 LMICs.
2. A global public health initiative to develop the needed manufacturing capability for complex (injectable) formulations. The current situation with CAB-RPV LA injectables show that holding the licence alone does not guarantee availability of generic products. The often-complex underlying technology constitutes major intellectual property of the innovator company. The technology that could deliver a novel LA formulation with biodistribution and pharmacokinetic profiles that are sufficiently similar to those of the innovator product is beyond the capacity of most generic manufacturers, both in terms of cost and expertise. One possible solution is a global public health initiative to develop the needed manufacturing capability for complex formulations like LA therapeutics. The goal should be to de-risk the landscape for both innovators and generic manufacturers, the latter focusing on LMIC markets. This will remove the likelihood that long-running safety studies will be required for regulatory approval of generic LA products due to the use of a novel underlying technology.
3. *In silico* bioequivalence studies. A demonstration of bioequivalence with the innovator LA formulation is a primary prerequisite for approval. The United Stated Food and Drug Administration and Control (US FDA) supports the use of *in silico* models to supplement partial AUC obtained from abridged (2-week) pharmacokinetic studies (156). This removes potential obstacles associated with conducting long-running bioequivalence studies for generic LA products which delay access in LMICs.
4. A mechanism to support generic manufacturers in navigating the regulatory pathways and opportunities to shorten it would be hugely impactful. This will expedite progress in realising the full potential of LA therapeutics in tackling infections of global health importance.
5. Technology to support co-delivery of multiple anti-infective compounds used in combination. Many pathogens require multiple drugs in combination to targeting two or more viral proteins. Such combination products as the DcNP systems being developed for HIV or combination particle suspension being developed for HCV will simplify the programmatic aspects for successful introduction.

## Figures and Tables

**Table 1:** Some Notable Long-acting Therapeutics Approved or Currently in Development for Infections of Global Public Health Importance

Indication	Drug	Dosing Information	Enabling Technology/Formulation	Pivotal Studies^1^	Status^2^
HIV(Treatment/PrEP)	Cabotegravir and Rilpivirine	Monthly or every 2 months	Nanocrystal suspension	ATLAS (NCT02951052) ATLAS-2M (NCT03299049)	FDA approved
	Cabotegravir	Monthly or every 2 months	Nanocrystal suspension	ATLAS	FDA approved
	Cabotegravir ultra-long acting	Every 4 months	Nanocrystal suspension	NCT06741397	Phase 2
	Lenacapavir	Every 6 months	API dissolved in PEG300; drug crystal deposited at injection site	CAPPELLA	FDA approved
	Lenacapavir	Every 12 months	-	PMID:40086460	Phase 1
	Cabotegravir and Lenacapavir	Every 8 weeks (cabotegravir) and Every 24 weeks (lenacapavir) injectable	-	CALENDULA (NCT06657885)	Phase 1
	Islatravir and Lenacapavir	Weekly	Oral tablet	ISLEND-1 (NCT06630286)	Phase 3
**Dosing Information**	**Technology/Formulation**
				ISLEND-2 (NCT06630299)	
	Dapivirine	Every 4 weeks	Intravaginal ring	ASPIRE	Approved in 11 African countries
	Lopinavir, Ritonavir and Tenofovir	Monthly	Lipid nanoparticles suspension	TLC-ART 101	Phase 1
Hepatitis C	Glecaprevir and Pibrentasvir	Monthly	Aqueous nanoparticle suspension	PMID:9678383	Preclinical
Tuberculosis	Bedaquiline	Not yet established (ascending dose study ongoing)	Microsuspension	EUCT #: 2023­508810-41-00	Phase 1
	Rifabutin	Every 2 months	Biodegradable ester- capped poly (lactic-co- glycolic acid) polymer	PMID:35941109	Preclinical
	SnPPIX-TIF	Once weekly	Poloxamer-polyacrylic acid-based thermoresponsive injectable	PMID: 33615179	Preclinical
Malaria	CIS43LS	Single dose (dose-escalation study ongoing)	Monoclonal antibody	NCT04329104 NCT04206332	Phase 2
	DSM265	Once weekly	Oral tablet	NCT02450578 NCT02123290 NCT02562872 NCT02573857	Phase 2a
	L9LS	6-Monthly seasonal prophylaxis	Monoclonal antibody	NCT05304611 NCT05816330 NCT05019729 NCT05400655	Phase 2
	MMV371	3-Monthly seasonal prophylaxis	Self-emulsifying drug delivery injectable	NCT06558643	Phase 1
	Ivermectin	Once fortnightly (Oral) 6-Monthly injectable	Oral tablet Injectable	ACTRN1262100121 8886	Phase 1 (Oral) Preclinical (Injectable)
	Artemether & Lumefantrine	Not yet established	Microarray patch	PMID:33794272	Preclinical
	Atovaquone	Once monthly	Solid drug nanoparticle Injectable	PMID:29358624	Preclinical

1 Pivotal Studies: clinical trial acronym, clinical trial registry ID (e.g. clinicaltrials.gov ID), or PMID number if already published

2 Status: Wherever there are multiple development stages for a drug, the most advanced stage is indicated. Abbreviations: SnPPIX-TIF, aqueous tin protoporphyrin IX (SnPPIX) loaded poloxamer-poly(acrylic acid)-based thermoresponsive injectable formulation.

## Terms and Definitions List

API: Active pharmaceutical ingredient
ART: Antiretroviral therapy
bNAbs: Broadly neutralizing antibodies
DAAs: Direct-acting antiviral
DcNP: Drug Combination Nanoparticulate
GRS: Gastric resident systems
HBV: Hepatitis B
HCV: HepatitisC
INSTI: Integrase strand transfer inhibitor I
RR: Incidence rate ratio
LA: Long-acting
LAI: Long-acting injectable
MAP: Microarray patch
MDR TB: Multidrug-resistant tuberculosis
mAbs: Monoclonal antibodies
*Mtb*: Mycobacterium tuberculosis
NHP: Non-human primates
PBPK: Physiologically-based pharmacokinetic
PrEP: Pre-exposure prophylaxis
